# Artificial intelligence-based PET denoising could allow a two-fold reduction in [^18^F]FDG PET acquisition time in digital PET/CT

**DOI:** 10.1007/s00259-022-05800-1

**Published:** 2022-05-20

**Authors:** Kathleen Weyts, Charline Lasnon, Renaud Ciappuccini, Justine Lequesne, Aurélien Corroyer-Dulmont, Elske Quak, Bénédicte Clarisse, Laurent Roussel, Stéphane Bardet, Cyril Jaudet

**Affiliations:** 1 Department of Nuclear Medicine, Baclesse Cancer Center, 3 Avenue Général Harris, 14076 Caen, France; 2 Department of Clinical Research, Baclesse Cancer Center, 3 Avenue Général Harris, 14076 Caen, France; 3 Department of Radiophysics, Baclesse Cancer Center, 3 Avenue Général Harris, 14076 Caen, France; 4 GIP CYCERON, Normandie Univ, UNICAEN, CNRS, ISTCT Unit, Caen, France; 5 Department of Informatics, Baclesse Cancer Center, 3 Avenue Général Harris, 14076 Caen, France

**Keywords:** [^18^F]FDG, PET, Denoising, Artificial intelligence, Deep learning, Acquisition time

## Abstract

**Purpose:**

We investigated whether artificial intelligence (AI)-based denoising halves PET acquisition time in digital PET/CT.

**Methods:**

One hundred ninety-five patients referred for [^18^F]FDG PET/CT were prospectively included. Body PET acquisitions were performed in list mode. Original “PET90” (90 s/bed position) was compared to reconstructed ½-duration PET (45 s/bed position) with and without AI-denoising, “PET45AI and PET45”. Denoising was performed by SubtlePET™ using deep convolutional neural networks. Visual global image quality (IQ) 3-point scores and lesion detectability were evaluated. Lesion maximal and peak standardized uptake values using lean body mass (SUL_max_ and SUL_peak_), metabolic volumes (MV), and liver SUL_mean_ were measured, including both standard and EARL_1_ (European Association of Nuclear Medicine Research Ltd) compliant SUL. Lesion-to-liver SUL ratios (LLR) and liver coefficients of variation (CV_liv_) were calculated.

**Results:**

PET45 showed mediocre IQ (scored poor in 8% and moderate in 68%) and lesion concordance rate with PET90 (88.7%). In PET45AI, IQ scores were similar to PET90 (*P* = 0.80), good in 92% and moderate in 8% for both. The lesion concordance rate between PET90 and PET45AI was 836/856 (97.7%), with 7 lesions (0.8%) only detected in PET90 and 13 (1.5%) exclusively in PET45AI. Lesion EARL_1_ SUL_peak_ was not significantly different between both PET (*P* = 0.09). Lesion standard SUL_peak_, standard and EARL1 SUL_max_, LLR and CV_liv_ were lower in PET45AI than in PET90 (*P* < 0.0001), while lesion MV and liver SUL_mean_ were higher (*P* < 0.0001). Good to excellent intraclass correlation coefficients (ICC) between PET90 and PET45AI were observed for lesion SUL and MV (ICC ≥ 0.97) and for liver SUL_mean_ (ICC ≥ 0.87).

**Conclusion:**

AI allows [^18^F]FDG PET duration in digital PET/CT to be halved, while restoring degraded ½-duration PET image quality. Future multicentric studies, including other PET radiopharmaceuticals, are warranted.

**Supplementary Information:**

The online version contains supplementary material available at 10.1007/s00259-022-05800-1.

## Introduction

Recent research in PET has focused on decreasing noise and increasing signal-to-noise ratios (SNR) [1]. Digital PET with silicon photomultipliers (SiPM) has led to improved timing, energy, spatial resolution, and effective time-of-flight (TOF) sensitivity [2–5]. This has resulted in faster scanning with less injected activity [1]. However, despite these advances, there is an ever-increasing demand for PET scans, which can contribute to significant delays in scheduling examinations and patient management.

Deep learning (DL), a subdivision of artificial intelligence (AI), has many emerging applications in nuclear medicine [6, 7]. DL is able to increase PET resolution, decrease noise, and thus enhance image quality [8–12]. It may allow for reducing injected activity, acquisition time, or a combination of both [10, 13–21].

DL-based denoising can either be associated with PET reconstruction or be used as a post-reconstruction tool. SubtlePET™ (Subtle Medical, Stanford, US, provided by Incepto, France) is a post-reconstruction PET denoising software that has been approved by the Food and Drug Administration and validated by the European Commission for [^18^F]FDG PET [22]. The algorithm is based on deep convolutional neural networks (DCNN), the most common DL architecture [23, 24]. SubtlePET™ uses multi-slice 2.5D encoder-decoder U-Net DCNN. It takes the pixel’s neighborhood into account to reduce noise and increase image quality. Using a residual learning approach that is optimized for quantitative (L1 norm) and structural similarity (SSIM), it has learned to separate and suppress noise components while preserving non-noise components.

Recently, SubtlePET™ processed [^18^F]FDG PET images obtained with 33% less injected activity gave similar visual and quantitative performances to native PET in analog PET/CT without time-of-flight (TOF) [25]. Promising results were also reported while reducing reconstructed PET acquisition time by 75% using analog and digital PET/CT (with or without TOF) in a smaller study population with a substantially higher original time-activity PET product [26]. Our group demonstrated the stability of most [^18^F]FDG PET radiomics features while applying this software without study count reduction [27].

In this prospective study, we aimed to evaluate the feasibility of halving PET acquisition time in a routine clinical setting by using SubtlePET™ while preserving visual and semi-quantitative PET performances in digital TOF PET/CT.

## Materials and methods

### Patient selection

One hundred ninety-five adult patients referred to our comprehensive cancer center for initial or follow-up [^18^F]FDG PET/CT from end-January to end-February 2021 were prospectively included in this study. The only exclusion criterion was a specific acquisition protocol involving a longer acquisition time per bed position on the head and neck or liver areas.

This non-interventional clinical study was approved by the local institutional review board from the François Baclesse Comprehensive Cancer Center and was registered with the French Health Data Hub under reference N° F20210720123322 on 20 January 2021. All patients provided informed consent to the use of their data.

### Imaging protocol and processing

All exams were performed in accordance with the EANM imaging guidelines [28] on a digital SiPM PET/CT (VEREOS, Philips Healthcare). After a 6-h fasting period, patients were injected with 3 MBq/kg [^18^F]FDG intravenously.

Before each PET scan, a low-dose non-contrast-enhanced CT scan was acquired for attenuation correction and as an anatomical reference. CT scan parameters were 100–140 kV, with variable mAs according to a dose right index of 14 and an iterative reconstruction Idose of 4:64 × 0.625-mm slice collimation, the pitch of 0.83, rotation time 0.5 s, 3D modulation, matrix 512 × 512 and voxel size 0.97 × 0.97 × 3 mm^3^.

PET acquisition, 1 h post-injection, was recorded in list-mode. Its field comprised at least the skull base to the upper thigh and was extended to total body acquisition if needed. Two PET reconstructions were performed: one for routine clinical purposes using the full acquisition time of 90 s per bed position (“PET90”), and a second one using 45 s per bed position for the purpose of this study (“PET45”). For both reconstructions, we used 3D ordered subset expectation maximization (3D-OSEM) with Point Spread Function (PSF), 4 iterations with 4 subsets (4i4s), a 2 × 2 × 2 mm^3^ voxel size, and 288 × 288 matrix size. Scatter, attenuation, and random corrections were computed.

PET45 images were processed by SubtlePET™ and are referred to hereafter as “PET45AI.” A fully automatic workflow allowed image transfer as well as denoising. A common and affordable NVIDIA 1080 GPU processor was used for SubtlePET™.

### Image analysis

#### Visual analysis

Original blinded PET90 and PET45AI were reviewed side-by-side by five experienced nuclear medicine physicians on a Syngo.via viewing server (version VB 30A, Siemens Healthcare). Each reader interpreted a unique part of the study population (all images per patient) and did not review PET/CT scans they had previously seen in clinical practice.

Readers attributed a global, whole-image quality (IQ) score to each PET series: 1 = poor; 2 = moderate; 3 = good. It was based on global and hepatic image noise and on normal tissue contrast.

All lesions with increased [^18^F]FDG uptake were notified on each PET series. For each lesion, the readers specified the preferred PET series for detection (related to the contrast-to-background ratio), the supposed nature, i.e. malignant (primary tumor, local recurrence, (nodal) metastasis), benign or indeterminate, and its location.

Additionally, to evaluate the incremental value of AI-based denoising, PET45 was compared to PET90 and PET45AI in 146 patients (due to missing data).

#### Semi-quantitative analysis

Lesions were independently and semi-automatically segmented on each PET series, using the 50% 3D-isocontour of the maximal pixel value.

In each lesion volume-of-interest (VOI), the following standardized uptake values based on lean body mass (SUL) were measured: SUL_max_ and SUL_peak_. LBM was estimated using the Janma formula [29].

The metabolic volume (MV) of the lesion and, when feasible, its short and long axes on the associated CT were calculated.

In addition, the reference liver SUL_mean_ with its standard deviation (SD) were collected in a 3 cm-diameter VOI in the right liver lobe, identical for each PET series.

Both standard and EARL_1_ (European Association of Nuclear Medicine Research Ltd) SUL were analyzed. EARL_1_ SUL was obtained numerically by Gaussian post-filtering within Syngo.via (EQ.PET filter [30]), with a full width at half maximum (FWHM) of 7.2 mm for all PET series. Our center is EARL accredited, and we use EARL_1_ SUL in routine practice for quantification, as it is transposable to different PET cameras and reconstructions [31].

Lesion-to-liver ratios (LLR) were calculated as SUL/SUL_mean_ liver and the coefficient of variation in the liver (CV_liv_) as SD/ SUL_mean_.

### Statistical analysis

Shapiro–Wilk testing found all quantitative variables (except for denoising processing time and SUL differences) to be non-normally distributed, further expressed by the median and interquartile range (IQR).

IQ scores between two PET series were compared by the Wilcoxon signed-rank test with continuity correction for paired data. Concordance rates of lesion detection between PET90 and PET45AI and between PET90 and PET45 were compared by the chi-squared test. Differences in continuous quantitative variables (semi-quantitative PET measures) between two PET series were statistically analyzed by the Wilcoxon signed-rank test for paired data.

Intraclass correlation coefficients (ICC) between semi-quantitative measures in PET90 and PET 45AI were also calculated, considering 0.5–0.75 as moderate, 0.75–0.9 as good, and > 0.9 as excellent reliability [32]. Absolute differences in SUL between PET series were calculated as SUL_PET45AI_ − SUL_PET90_ and relative differences or delta ∆ as (SUL_PET45(AI)_ − SUL_PET90_) / SUL_PET90_, and likewise for MV.

Bland Altman plots were used to display absolute SUL differences between PET90 and PET45AI, with Limits of Agreement (LOA) computed as the mean difference ± 1.96 × SD. A logistic uni- and multivariable regression analysis was carried out to look for predictive factors of a decrease of over 10% in SUL_max_ in PET45AI vs PET90.

This decrease threshold of 10% was set by the required accuracy of SUL calibration within 10% for VEREOS PET, according to the AAPM report 126 [33]. Bonferroni correction for statistical significance level was used in univariable logistic regression analysis. Elsewhere, *P*-values < 0.05 were considered statistically significant. Analyses were conducted with R version 4.0.2.

## Results

### Patient population and image processing

The main characteristics of the 195 patients included in this study are shown in Table 1.

**Table 1:** Patient characteristics

*N* = 195	
Age (years), median; IQR^1^	66; 59–74
Gender, *n* (%)	
Male	122 (63)
Female	73 (37)
Weight (kg), median; IQR	72; 61–84
BMI ^2^ (kg/m^2^)	26; 23–30
Glycaemia (g/l)	1.05; 0.95–1.20
Scan delay (min) ^3^	57; 55–59
Study indication (n (%) of patients)	
*Malignancy*	147 (75)
Baseline staging	39 (20)
Therapeutic evaluation	65 (33)
Recurrence detection/staging	43 (22)
*Characterization (benign vs malignant)*	41 (21)
*Miscellaneous*	7 (4)
Primary lesion (origin)	
*Lung*	70 (36)
*Breast*	68 (35)
*Gynecological (except breast)*	17 (9)
*Colorectal*	6 (3)
*Lymphoma*	6 (3)
*Unknown primary*	6 (3)
*Sarcoma*	5 (3)
*Other* ^*4*^	23 (12)

^1^ *IQR,* interquartile range between first and third quartile (Q1 and Q3); ^2^ *BMI*, body mass index; ^3^ 2 patients had a delay < 55 or > 65 min pi.(53 and 70 min); ^4^ Other primaries: prostate (4), melanoma (3), head-and-neck (3), esophagus and stomach (3), bladder (3), testicle (1), pancreas (1), anus (1), myeloma (1), mesothelioma (1), skin squamous cell carcinoma (1), Merkel cell carcinoma (1). 12 patients had more than one primary; 7 patients had no primary (sum > 195)

All two-fold count reduced PET series (PET45) were successfully treated by the denoising software with a mean processing time of 90 s (min–max; 45–122 s).

### Image analysis

## Visual analysis: comparison between PET90 and PET45AI

IQ scores were similar between original PET90 and PET45AI (*P* = 0.80), good (score 3) in 92% (*n* = 180 vs 179) and moderate (score 2) in 8% (*n* = 15 vs *n* = 16) of exams.

Concerning lesion detection, 33 out of 195 patients presented a normal and concordant examination on both PET series. In the remaining 162 patients, a total of 856 lesions were detected.

**Fig. 1:**
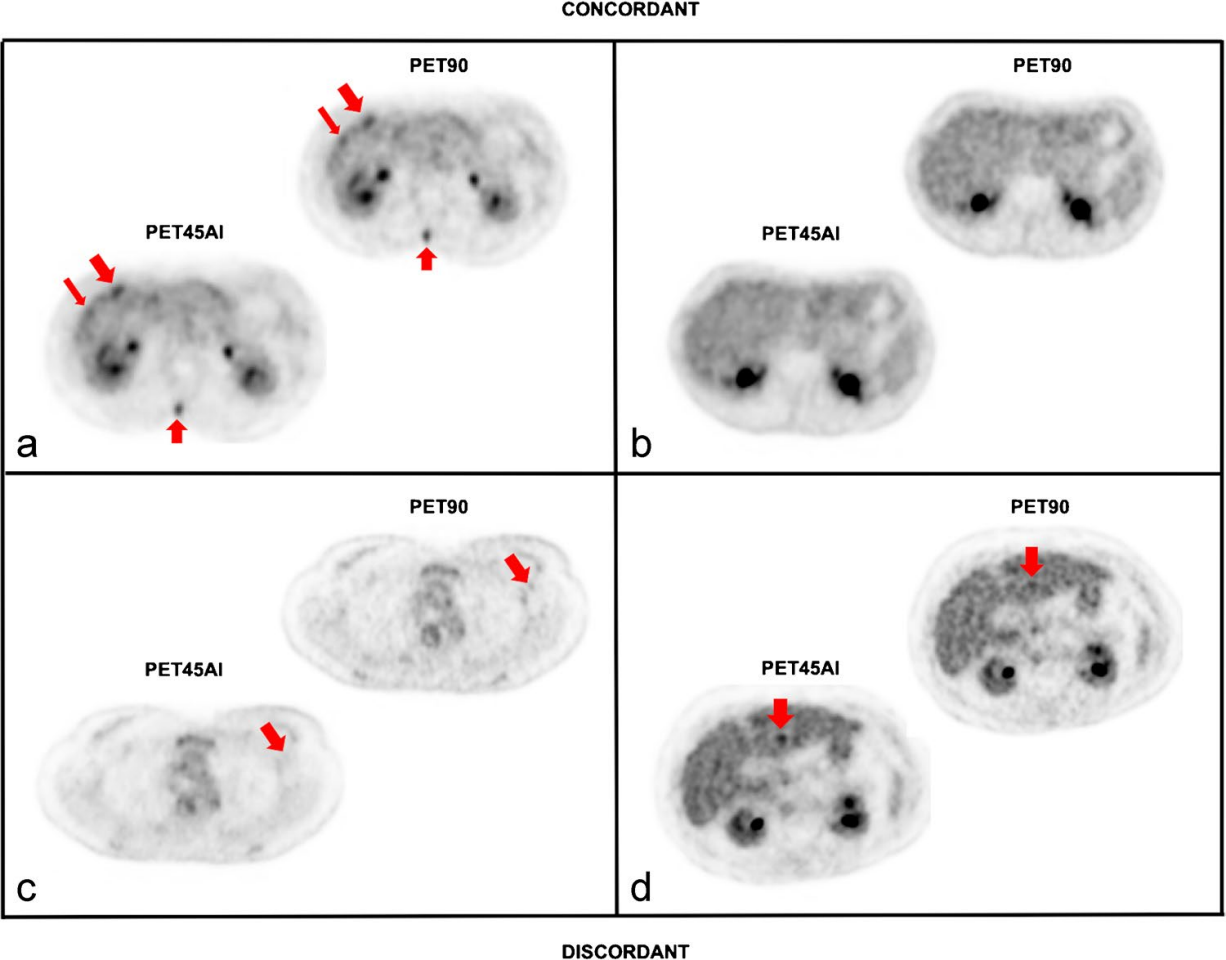
Two concordant and two discordant PET images between PET90 and PET45AI In **a** several hepatic (oblique red arrows) and a spinal bone metastasis (vertical upward red arrows) in a female patient with breast cancer were detected on both original PET90 and denoised PET45AI. In **b** a concordantly negative PET. In **c** a low-uptake, sub-centimetric left axillary lymph node (oblique red arrows) in a patient referred for left breast cancer staging, classified indeterminate and exclusively detected on original PET90. In **d** an indeterminate liver focus exclusively annotated on PET45AI (vertical downward red arrows) in a male patient scanned for advanced lung cancer staging

Of these, 836 lesions were visualized in both original PET90 and denoised PET45AI, resulting in a lesion concordance rate of 97.7%. Seven out of 856 (0.8%) small and low-uptake lesions were detected exclusively on PET90 in 6 patients (Table 2). Thirteen foci (1.5%) were detected only on PET45AI in 10 patients, mostly corresponding to indeterminate liver lesions. An illustration is shown in Fig. 1.

**Table 2:** Description of discordant lesions between PET90 and PET45AI

	Patient	Lesion	Malignancy	Location	Nature
Original PET90 only	1	1	lymphoma	retroperitoneal lymph node	malignant
	2	2	breast	lung	malignant (metastasis)
	3	3	ovarium	peritoneum	malignant (metastasis)
		4		peritoneum	malignant (metastasis)
	4	5	breast	bone	malignant (metastasis)
	5	6	breast	axillary lymph node	indeterminate
	6	7	lung	small intestine	indeterminate
PET45AI only	7	8	mesothelioma	liver	indeterminate
		9		liver	indeterminate
	8	10	hypopharynx	liver	indeterminate
	9	11	lung	liver	indeterminate
		12		liver	indeterminate
	10	13	breast	liver	indeterminate
	11	14	multiple myeloma	liver	indeterminate
	12	15	breast	liver	indeterminate
	13	16	lung	liver	indeterminate
	14	17	breast	liver	indeterminate
	15	18	breast	spleen	indeterminate
	16	19	colon	bone	benign
		20		bone	indeterminate

There was no per-lesion preferred PET series for detection in 86% of lesions. On the other hand, original PET90 was preferred for 12% and PET45AI for 2%.

## Semi-quantitative analysis: PET90 and PET45AI measures

### Statistical comparison of standard values

Lesion SUL_max_, SUL_peak_, LLR, and CV_liv_ were significantly lower in denoised PET45AI than in original PET90 (*P* < 0.0001) (Table 3). In contrast, lesion MV and liver SUL_mean_ were higher in PET45AI than in PET90 (*P* < 0.0001). Lesion SUL, MV, LLR, and liver SUL_mean_ showed a good-to-excellent correlation between both PET series (≥ 0.873 up to 0.998).

**Table 3:** Standard semi-quantitative measures in original PET90 and denoised PET45AI

		PET90	PET45AI	ICC [95%CI]
Lesion	SUL_max_ (g/ml)	4.45 [3.08–7.53]	3.99 [2.68–6.94]	0.99 [0.98–0.99]
	SUL_peak_ (g/ml)	2.72 [1.87–4.77]	2.63 [1.79–4.65]	1.00 [0.99–1.00]
	MV (ml)	1.22 [0.61–2.90]	1.45 [0.80–3.30]	0.97 [0.97–0.98]
Liver	SUL_mean_ (g/ml)	1.66 [1.52–1.84]	1.77 [1.60–1.96]	0.87 [0.84–0.90]
	CV (%)	12.83 [11.71–14.49]	10.96 [9.55–12.41]	0.58 [0.48–0.66]
Lesion/liver	LLR_max_	2.68 [1.81–4.58]	2.32 [1.51–4.04]	0.97 [0.97–0.98]
	LLR_peak_	1.67 [1.12–2.86]	1.51 [1.01–2.69]	0.99 [0.99–0.99]

All values are expressed as median [interquartile range]. *ICC*, intraclass correlation coefficients between PET90 and PET45AI measures; *MV*, metabolic volume; *CV*, coefficient of variation; *LLR*, lesion-to-liver ratio

### Statistical comparison of EARL_1_values

Lesion EARL1 SUL_peak_ was not significantly different between both PET series (*P* = 0.09). Otherwise, the comparison of EARL1 SUL and derived measures between PET90 and PET45AI was similar to the comparison of standard measures.

### Absolute and relative differences

**Fig. 2:**
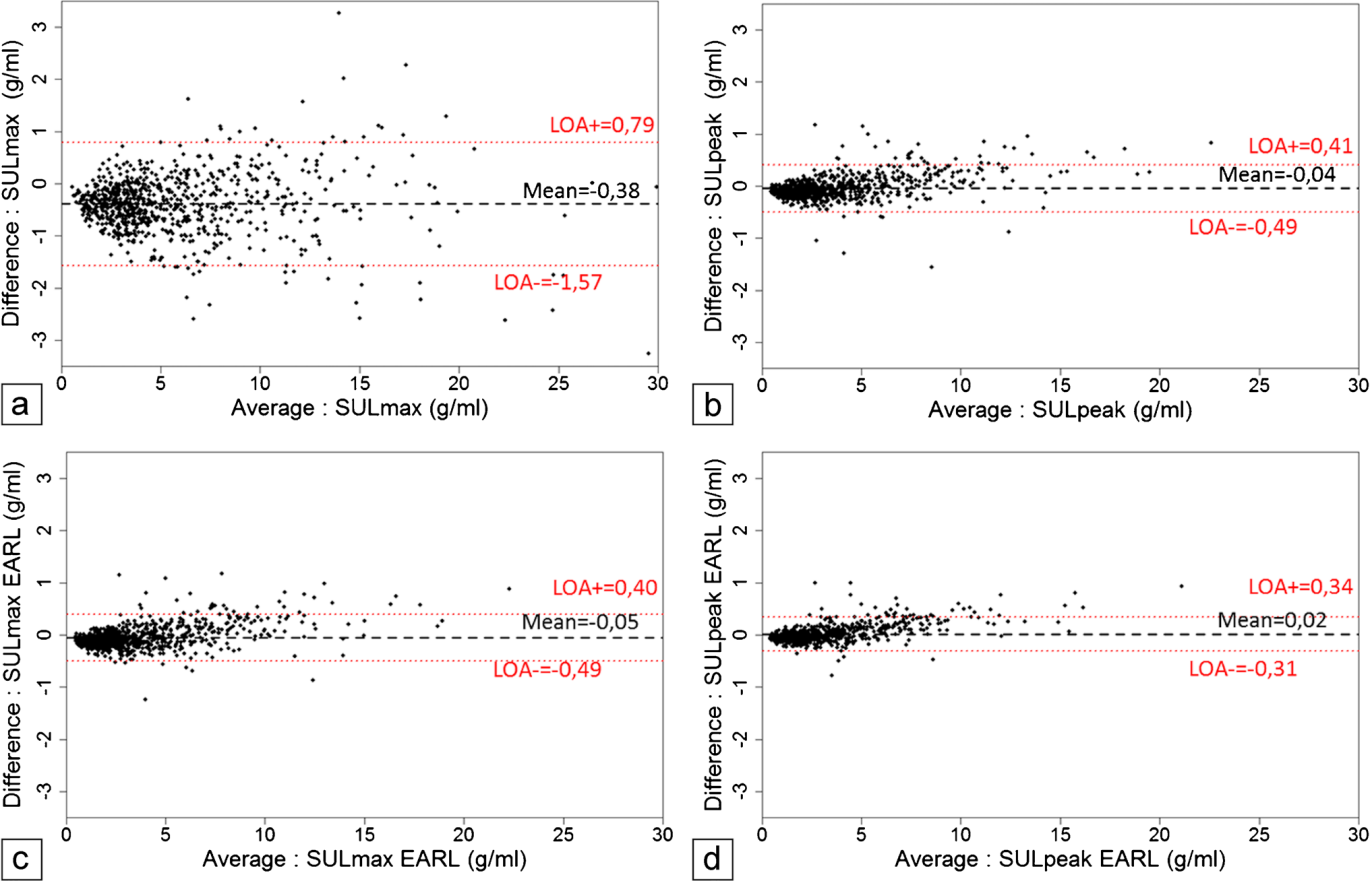
Bland Altman plots for standard SUL_max_ (**a**) and SUL_peak_ (**b**) and respective EARL_1_ SUL_max_ (**c**) and SUL_peak_ (**d**): *Y*-axis shows the absolute differences between PET45AI and PET90 SUL measures versus their means on *X*-axis. A dashed black line corresponds to the mean and dotted red lines to the upper and lower limits of agreement (LOA). Most lesions had SUL below 5 g/ml

Bland Altman (Fig. 2) plots show the absolute difference between both PET series in SUL_max_ and SUL_peak_ (both standard and EARL_1_ measures) for each lesion. The highest mean absolute difference reached − 0.38 g/mL [95% CI − 0.43, − 0.34] for standard SUL_max_ in PET45AI vs original PET90_._ The other average absolute differences were close to 0.

The mean ± SD relative differences in PET45AI compared to PET90 reached − 9.48 ± 11.50% for standard SUL_max_, − 3.41 ± 7.17% for standard SUL_peak_, − 3.74 ± 7.34% for EARL_1_ SUL_max,_ and − 1.37 ± 5.71% for EARL_1_ SUL_peak_ of lesions. For liver SUL_mean_, the mean relative difference was + 5.64 ± 4.75% and 5.88 ± 3.93% for standard and EARL_1_ measures, respectively.

## Explanatory factors analysis of differences between PET90 and PET45AI

### In visual lesion detection

Table 4 shows lesion characteristics (size and uptake) according to their detectability. Most discordant and preferred lesions had a low-to-moderate uptake and size.

**Table 4:** Lesion features according to their detectability in original PET90 and denoised PET45AI

	Size on CT (mm)		MV (ml) ^1^	SUL_max_ (g/ml)
***Absolute detection***	Long axis	Short axis		
**Concordant**	16 [10–24]	10 [7–16]	1.3 [0.6–3.2]	4.4 [3.1–7.5] ^(2)^
**Discordant **PET90 only	5 [5–6]	4 [3–6]	0.8 [0.6–1.2]	2.1 [1.5–2.6]
**Discordant **PET45AI only	NA^3^	NA	1.2 [0.6–2.9]	3.0 [2.5–3.3]
***Preferred serie for detection***				
**No**	17 [11–25]	11 [7–17]	1.5 [0.8–3.8]	4.6 [3.1–7.7] ^(2)^
**PET90**	9 [7–12]	7 [5–9]	1.1 [0.7–1.8]	2.7 [2.0–3.6] ^(2)^
**PET45AI**	8 [5–22]	6 [4–16]	1.5 [0.9–2.7]	2.2 [1.6–2.8] ^(2)^

All measures are displayed as median [interquartile range]; ^1^ *MV*, metabolic volume. Note that metabolic volumes of small lesions and with low contrast-to-background ratios are less accurate. ^2^ of original PET90; ^3^ *NA*, not assessable (no measurable CT lesion)

### In lesion SUL_max_

Multivariable logistic regression analysis indicated two independent predictors of a SUL_max_ decrease of over 10% in PET45AI compared to PET90, namely SUL_max_ in PET45AI (*P* < 0.0001) and CT long axis (*P* = 0.01) (Table 5). Supplementary Fig. 3 shows that the smaller the lesion size on CT and the lower the SUL_max_, the greater the probability of a negative SUL_max_ bias over 10%.

**Table 5:** Uni-and multivariable logistic regression analysis for predicting a negative ΔSULmax above 10% in PET45AI compared to PET90

	Univariable		Multivariable	
	OR [95% CI]	*P*	OR	*P*
Age	0.99 [0.82–1.21]	0.98	1.04 [0.81–1.34]	0.77
Female sex	1.87 [1.26–2.78]	0.002*	1.11 [0.65–1.87]	0.71
BMI	1.20 [0.99–1.44]	0.05	1.02 [0.78–1.32]	0.91
Glycaemia	1.18 [0.97–1.43]	0.10	1.17 [0.89–1.54]	0.25
SUL_max_^(1)^	0.18 [0.13–0.25]	< 0.0001*	0.22 [0.14–0.33]	< 0.0001*
CT long axis	0.20 [0.14–0.29]	< 0.0001*	0.49 [0.28–0.84]	0.01*
MV ^(1)^	0.06 [0.03–0.13]	< 0.0001*	0.28 [0.07–1.11]	0.07
CV_liv_^(1)^	0.98 [0.81–1.18]	0.81	1.35 [0.87–2.09]	0.18
CV_liv__Ratio	0.96 [0.82–1.14]	0.67	0.79 [0.56–1.11]	0.17

^*^ statistically significant. ^1^ of PET45AI. *OR*, odds ratio; *BMI*, body mass index; *MV*, metabolic volume; *CV*_*liv*_, coefficient of variation in the liver. CV_liv__Ratio = CV_liv_ (PET45AI) / CV_liv_ (PET90). PET45AI SUL_max_ values were used to build a predictive model focusing on the end result, namely denoised and not original PET. However, we obtained the same results with original PET90 SUL_max_. A negative ΔSULmax above 10% concerned 383 lesions (46%). Few lesions showed an increase above 10% in SUL_max_ (*n* = 9; 1.0%) on PET45AI vs PET90, not further analyzed

## Evaluation of PET45

### Visual analysis

**Fig. 3:**
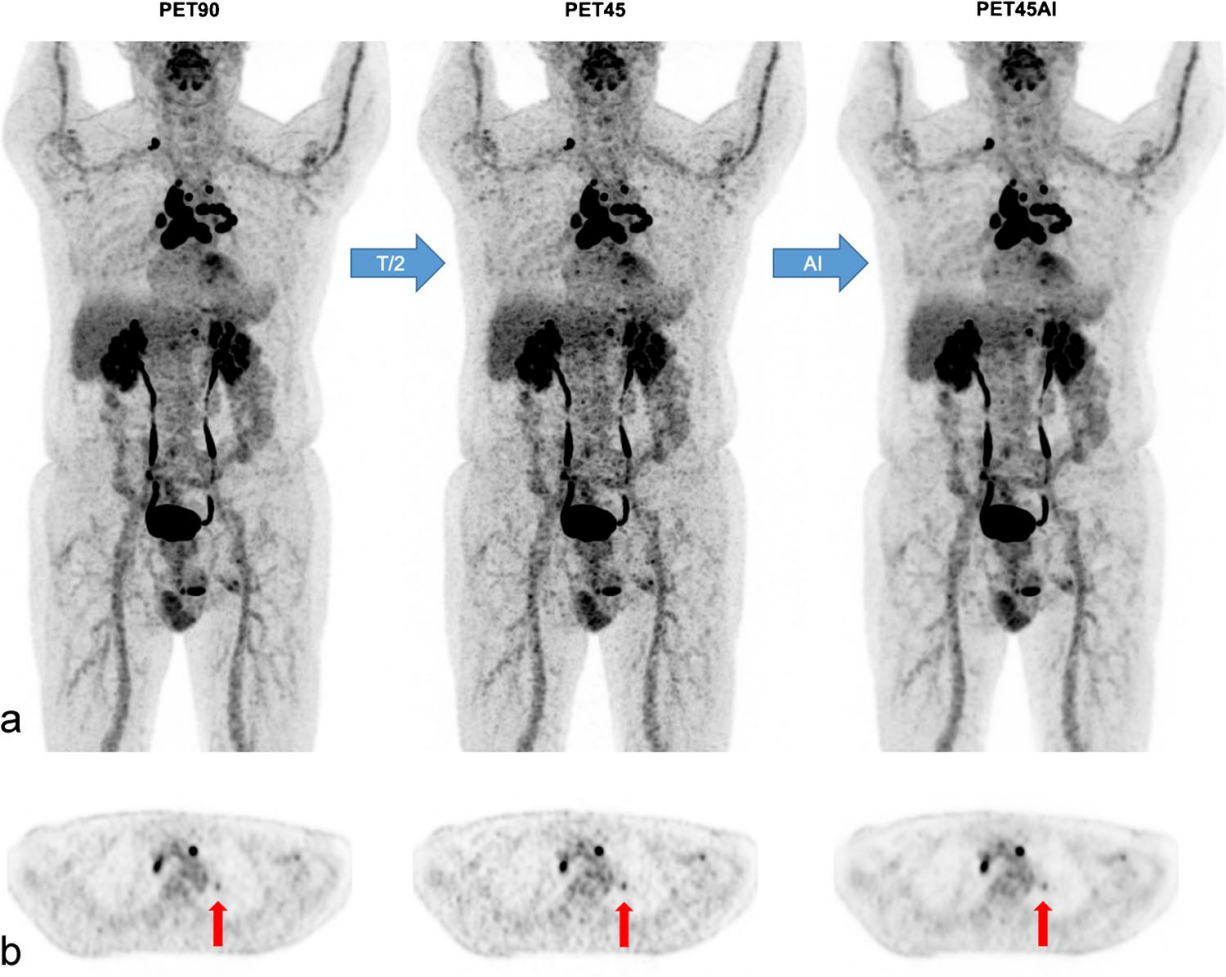
Concordant lesions A 77-year-old man (78 kg; BMI 24 kg/m^2^) with multifocal lymphadenopathy of unknown origin. MIP views (**a**) and axial PET slices (**b**) of [^18^F]FDG PET90, PET45, and PET45AI. Detection of small left suprahilar lymphadenopathy in all PET series (vertical arrows in b) with respective standard SUL_max_ of 1.8 (PET90), 2.3 (PET45), and 1.7 g/ml (PET45AI). Nonetheless, PET45 images are noisier than PET90 or PET45AI images, particularly in the liver

**Fig. 4:**
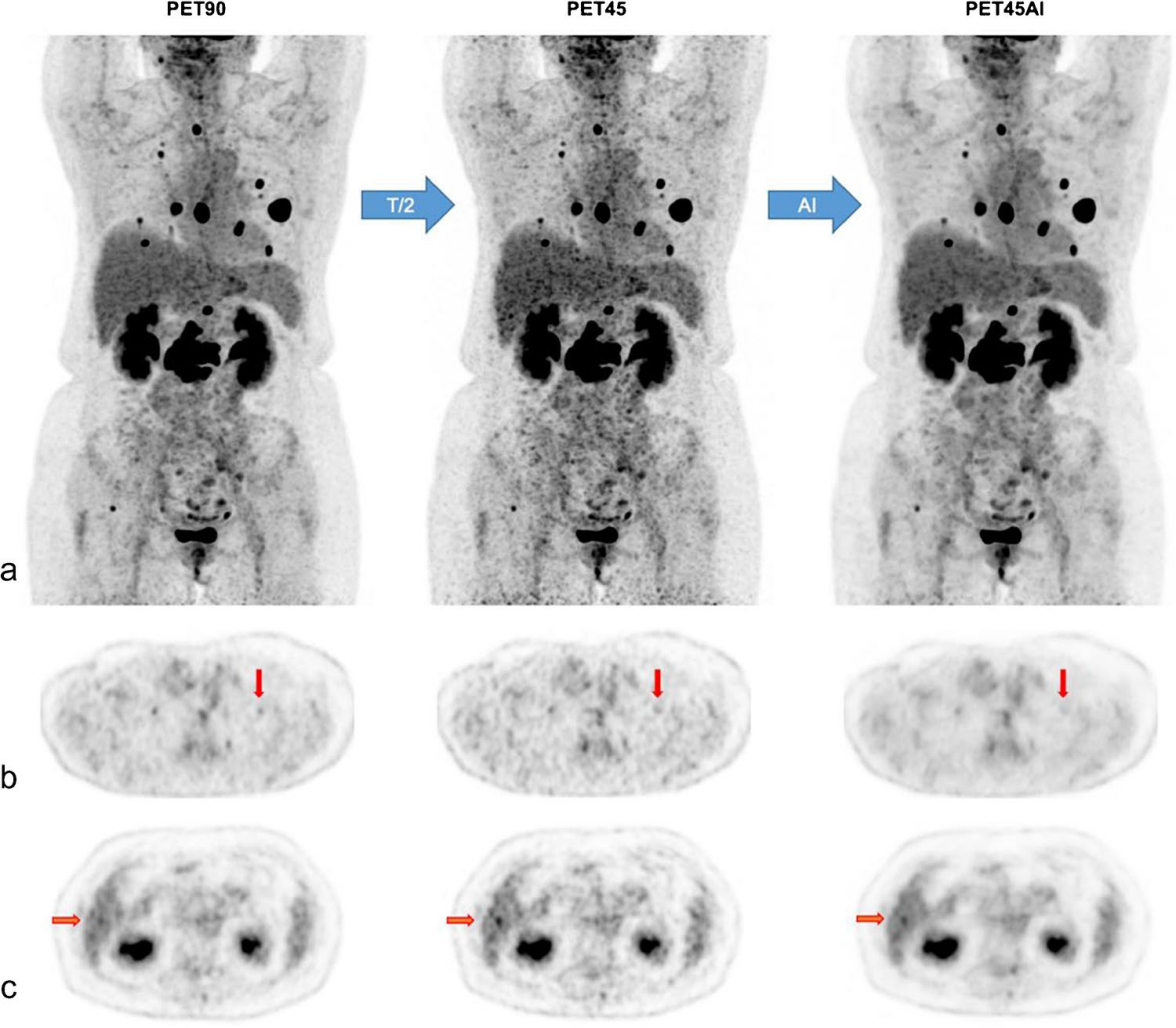
Discordant lesions A 59-year-old women (66 kg; BMI 23 kg/m^2^) with a history of breast cancer showing multiple lung and bone metastases. MIP views (**a**) and axial PET slices (**b** and **c**) of [^18^F]FDG PET90, PET45, and PET45AI. Vertical red arrows in (**b**) demonstrate one lung metastasis in the upper lobe of the left lung only detected in PET90, measuring 2 × 3 mm on CT with standard SUL_max_ of 1.1 g/ml in PET90. In **c** a false positive hepatic focus in PET45 (horizontal red arrows)

PET90, PET45, and PET 45AI were compared in 146 patients. Two cases are illustrated in Figs. 3 and 4.

IQ scores were lower in PET45 (median: 2) than in both PET90 and PET45AI (median: 3), *P* < 0.0001. Poor IQ scores (= 1) were exclusively found in PET45 scans (*n* = 12; 8%). IQ was scored moderate (= 2) in 99 (68%) PET45 examinations vs in 13 (9%) PET90 and 16 (11%) PET 45AI, the remainder being considered of good image quality.

In this subgroup of patients, the lesion detection concordance rate between PET90 and PET45 was 88.7% (582/656), while that between PET90 and PET45AI was 97.4% (589/605), *P* < 0.0001. The number of false-positive foci was higher in PET45 (*n* = 61; 9.3%) than in PET45AI *(n* = 10; 1.7%), *P* < 0.0001. Furthermore, 13 (2.0%) false-negative lesions were present in PET45 and 6 (1.0%) in PET45AI, *P* = 0.15.

### Semi-quantitative analysis

Lesion standard SUL_max_ was significantly higher in PET45 than in PET90 (*P* ≤ 0.0001, with an average ± SD relative bias of + 3.30 ± 10.34%). Lesion standard SUL_peak_, EARL1 SUL_peak_ and EARL1 SUL_max_ were similar in PET90 and PET45.

CV_liv_ was significantly higher in PET45 (median 18.00; IQR 15.98–21.16%) than in PET90 (12.84; 11.88–14.27%) and than in PET45AI (10.80; 9.68–12.21%), *P* < 0.0001.

## Discussion

This prospective study shows good visual and semi-quantitative performances of AI-denoised half-count PET compared to original PET in a digital PET/CT. We simulated a two-fold reduction in the PET acquisition time and then applied a commercially available PET denoising software based on U-net DCNN. All PET series were successfully denoised within 2 min in an automatic workflow using a common GPU card. This makes it compatible with routine clinical use. Visually, global image quality scores were similar between PET90 and PET45AI but lower and clinically insufficient in half-count PET45 due to high noise. We obtained few discordances (2.3%) between original PET90 and denoised PET45AI in the absolute detection of 856 lesions.

A total of 0.8% of lesions were detected only on PET90 in 3% of patients. This concerned sub-centimetric or small lesions with a maximum SUL_max_ of 3.1 g/ml. Most of these “original PET90-only or false-negative lesions in PET45AI” were classified as authentically malignant (71%) or indeterminate (29%). Many other concordant malignant lesions were detected in all but one of these patients.

A total of 1.5% of lesions were exclusively visualized on denoised PET45AI in 5% of patients. These “false positives” were predominantly located in the liver and interpreted as indeterminate or benign foci. For most lesions, there was no per-lesion preferred PET series for detection. However, in a minority of lesions (12%), original PET was preferred and less frequently (in 2%) denoised PET. Whether on original or on denoised PET, preferred lesions showed a variable uptake and size, mostly low-to-moderate. More expertise in the reading of these new denoised PET images could further improve the accuracy and comfort of readers.

A higher lesion detection discordance rate (> 10%) was found between PET90 and half-duration PET45 than between PET90 and PET45AI, with particularly additional false positives in PET45. This further renders half-count PET not compatible with routine clinical use. Similar results were observed in [21], with also a decrease in diagnostic confidence when dividing acquisition time by two.

Comparing semi-quantitative SUL measures in lesions between PET90 and PET45AI, only harmonized EARL_1_ SUL_peak_ was not significantly different when using the same Gaussian post-filter for both PET series. Standard SUL_peak_ and standard and EARL_1_ SUL_max_ were lower in denoised PET45AI than in original PET90. The average relative difference remained below 10% for all lesion SUL. Greater SUL biases occurred especially in lesions with a moderate size and uptake and mostly “non-target and non-evaluable lesions” according to PERCIST criteria [34, 35]. In our quantitative study, all lesions were taken into account. The overrepresentation of small, low-uptake lesions negatively affected quantitative differences between both PET series.

On the other hand, SUL_mean_ in the reference liver was slightly higher (on average + 6%) in PET45AI than in the original PET90. Its standard deviation and thus its noise levels were lower (on average − 12% for standard CV_liv_). The decrease in CV_liv_ highlights the denoising efficacy even when dividing study counts by two.

Some other research groups have found even lower SUV biases, despite a higher study count reduction, especially while using CycleGANs as DL architecture [16, 17] or Subtle PET™ (U-net) [26]. However, their studies were performed on different and/or smaller cohorts.

A pilot study of 10 small lung nodules suggested that a fully 3D U-net compared to a 2.5D U- net, as used in our study, may offer better lesion quantitative performance, even though visual image quality was similar [19]. However, 2.5D U-net is useful for routine clinical practice owing to its shorter computational time and lower processing capacity requirement.

Nevertheless, probably more important than these differences in semi-quantitative measures was their correlation between original PET90 and PET45AI, in particular for lesion SUL.

This inter-PET correlation was very high for lesion SUL and MV (with ICCs of at least 0.97) and high for liver SUL_mean_ (with ICCs of at least 0.87), testifying to the stability and reliability of these measures obtained after PET count reduction and denoising.

A strength of our study is a large number of lesions of very different sizes, uptake, nature, and location.

Study limitations are the side-by-side reading methodology which could have enhanced the detection accuracy in PET45(AI). Second, the clinical impact of denoised PET has not been properly established. Third, the unlimited lesion number per patient led to a potential statistical bias due to the over-representation of dependent lesions in the same patients. Fourth, the effect of AI-denoising on image artifacts was not studied. A final small drawback is a use of harmonized EARL_1_ SUL measures, which are still widely used, and not more recent EARL_2_ values [36].

Our study thus supports the routine use of Subtle PET™ combined with a two-fold faster PET acquisition.

The benefit of decreasing PET duration, thus reducing waiting time for appointments and helping patients who experience discomfort, outweighs the minor decrease in performance.

Although not properly studied, our findings could also lead to a reduction in injected activity or a combination of both (activity and time). Initially, an Italian group reported a similar performance of Subtle PET™ treated PET with 33% less injected [^18^F]FDG activity compared to native PET in non-TOF analog PET/CTs [25].

Further research should be carried out on ways to increase performances, e.g. by optimizing the DL-model and/or adapting acquisition time in liver and regions of interest. Furthermore, large multicentric studies with different PET cameras, reconstruction parameters, and various reductions in [^18^F]FDG PET acquisition time-activity product are necessary. Striking the optimal balance between performance and time savings is essential. Moreover, research with other PET radiopharmaceuticals is warranted.

## Conclusion

This prospective study demonstrates the satisfactory preservation of [^18^F]FDG PET image quality and quantification when applying AI-based denoising on half-duration PET compared to original full-duration PET. AI restored degraded and clinically insufficient image quality of half-duration PET. It paves the way for a significant reduction in acquisition time and the optimization of PET imaging equipment in routine clinical practice.

## Supplementary Information

Below is the link to the electronic supplementary material.

Supplementary file1 (PDF 98 kb)

## Data Availability

The datasets generated during and/or analyzed during the current study are available from the corresponding author on reasonable request.
