# Supplementary figures and images for: Artificial intelligence-based PET denoising could allow a two-fold reduction in [18F]FDG PET acquisition time in digital PET/CT
Source: Eur J Nucl Med Mol Imaging. 2022 May 20;49(11):3750–60. doi: 10.1007/s00259-022-05800-1 (PMC9399218; doi:10.1007/s00259-022-05800-1)

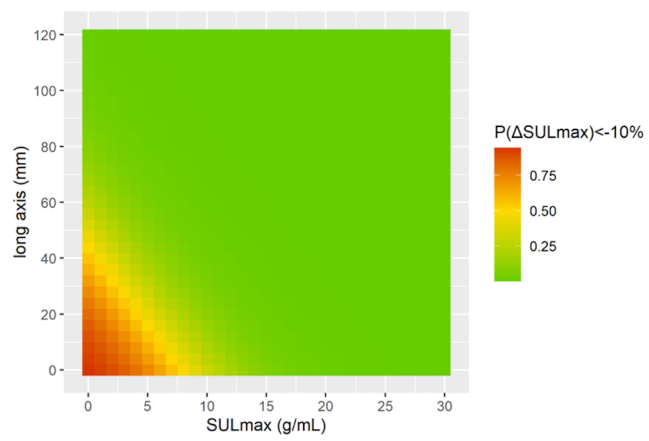

Supplement: Supplementary file 1 — Supplementary file1 (PDF 98 kb) [file 259_2022_5800_MOESM1_ESM.pdf]
